# Pilot–Scale Production of Carbon Hollow Fiber Membranes from Regenerated Cellulose Precursor-Part I: Optimal Conditions for Precursor Preparation

**DOI:** 10.3390/membranes8040105

**Published:** 2018-11-13

**Authors:** Shamim Haider, Jon Arvid Lie, Arne Lindbråthen, May-Britt Hägg

**Affiliations:** Department of Chemical Engineering, Norwegian University of Science and Technology (NTNU), 7491 Trondheim, Norway; haider@ntnu.no (S.H.); jonarvidlie@gmail.com (J.A.L.); arne.lindbrathen@ntnu.no (A.L.)

**Keywords:** pilot scale process, carbon membrane precursor, dry/wet spinning, deacetylation, regenerated cellulose, drying conditions

## Abstract

Industrial scale production of carbon membrane is very challenging due to expensive precursor materials and a multi-step process with several variables to deal with. The optimization of these variables is essential to gain a competent carbon membrane (CM) with high performance and good mechanical properties. In this paper, a pilot scale system is reported that was developed to produce CM from regenerated cellulose precursor with the annual production capacity 700 m^2^ of CM. The process was optimized to achieve maximum yield (>95%) of high quality precursor fibers and carbonized fibers. A dope solution of cellulose acetate (CA)/Polyvinylpyrrolidone (PVP)/*N*-methyl-2-pyrrolidone (NMP) and bore fluid of NMP/H_2_O were used in 460 spinning-sessions of the fibers using a well-known dry/wet spinning process. Optimized deacetylation of spun-CA hollow fibers (CAHF) was achieved by using 90 vol% 0.075 M NaOH aqueous solution diluted with 10 vol% isopropanol for 2.5 h at ambient temperature. Cellulose hollow fibers (CHF) dried at room temperature and under RH (80% → ambient) overnight gave maximum yield for both dried CHF, as well as carbon fibers. The gas permeation properties of carbon fibers were also high (CO_2_ permeability: 50–450 Barrer (1 Barrer = 2.736 × 10^−9^ m^3^ (STP) m/m^2^ bar h), and CO_2_/CH_4_ selectivity acceptable (50–500).

## 1. Introduction

In the 1960s, the term carbon membrane was introduced for the first time for carbon plugs prepared from compressed carbon powders [1,2]. However, the materials reported were too porous to achieve selective membranes. Two decades later Koresh and Soffer [3] reported the concept of pre-shaped polymeric precursor materials for the direct formation of carbon membranes. In 1995, Soffer et al. [4] developed and patented the protocol for carbonization of cellulose precursor. Cellulose is a natural polymer that produced by plants through photosynthesis. It is abundantly available in the world. However, only a limited number of solvents exist to dissolve (strong inter and intra-molecular hydrogen bonding [5]) and treat cellulose in such way that monosaccharides are preserved to form a suitable carbon structure after carbonization [6,7].

Cellulose esters, in particular, cellulose acetate (CA), is relatively inexpensive, easily commercially available, and have been applied successfully for many years, as a membrane material in different applications, such as water treatment, pervaporation, gas separation etc. [8,9,10,11,12]. CA is soluble in common solvents like NMP (*N*-methyl-2-pyrrolidone), DMSO (dimethylsulfoxide) and acetone. However, direct carbonization of CA will result in discontinuous carbon (more like a powder), since the intermediate product levoglucosane [7] is not formed during carbonization. Hence, CA must be deacetylated after the membrane casting/spinning process to allow the formation of the critical intermediate product of levoglucosane.

Deacetylated cellulose hollow fibers (CHF) from spun-cellulose acetate hollow fibers (CAHF) showed good potential to as a precursor for carbon hollow fiber membrane (CM) with promising permeation properties in gas separation applications when tested on laboratory scale [13,14,15,16,17]. However, the scaling up process and control of spinning parameters for CAHF, post-treatment of the spun fibers, deacetylation to obtain CHF, and drying of regenerated CHF have been challenging processes to obtain the carbon fibers with good mechanical and acceptable permeation properties.

**Figure 1:**
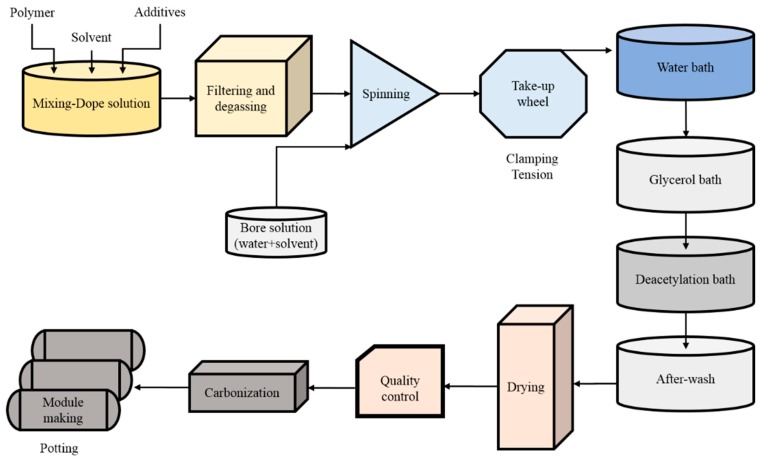
Block diagram for the pilot-scale production process of carbon membrane (CM) from cellulose acetate hollow fibers (CAHF).

The goal for and novelty of this study was to develop and optimize a pilot-scale production process for the CHF, using a low-priced precursor to generate carbon membranes. The sub-goal for the development of the pilot-scale facility was to achieve CM exhibiting high permeability and selectivity for the CO_2_/CH_4_ separation for biogas upgrading to vehicle fuel or for natural gas purification (natural gas sweetening). The separation properties of the CM for O_2_/N_2_ separation were also investigated. The CM from CHF were produced and tested on semi-commercial scale plant by MemfoACT AS (Norway), a company which has now closed down. The plant had a capacity to produce 700 m^2^ of carbon hollow fiber membranes per year. The well-known dry/wet spinning method was used to spin the CAHF [18,19] from the dope solution consisting of CA/polyvinylpyrrolidone (PVP)/*N*-methyl-2-pyrrolidone (22.5%/5%/72.5% *w*/*w*/*w*). The spinning parameters, such as dope and bore fluid composition, extrusion rate, spinneret dimensions, air gap, coagulation temperature, take up rate and solvent exchange method, were optimized to achieve maximum yield (based on length, quantity of good fibers, mechanical properties of CHF, and separation properties of resulting CM) of CHF. The CAHF can be deacetylated by exchange of acetyl group with a hydroxyl group using a base catalyst [12]. Various authors have reported the importance of different types of base solutions, concentrations, and the reaction time for deacetylation process [12,20,21,22,23]. The deacetylation process is crucial to regenerate CHF with desired structure and properties as a precursor for carbon membrane. However, not much data is available for the pilot-scale deacetylation of CAHF. The deacetylation of CAHF was in our study performed by using NaOH/water/isopropanol solution and different parameters were optimized to enhance the yield of dried precursor CHF. A block diagram for the pilot-scale production of CM from CAHF is shown in Figure 1. As shown in Figure 1, the next challenging step after deacetylation is after-wash/solvent rinsing and drying of the CHF.

The resulting cellulose fibers are more hydrophilic than CAHF and stronger hydrogen bonds are expected between cellulose and water. An efficiently controlled drying process was thus needed to dry these membranes. The natural drying of these membranes at ambient conditions influenced the structure (shrunken, curly, adhered, and/or collapsed fibers) and permeation properties of resulting carbon membranes.

Jie et al. [24] investigated natural drying and solvent exchange drying methods and concluded that ethanol-hexane exchange drying was an appropriate method to produce a membrane with minimum morphology variation. Whereas the natural drying caused the greatest shrinkage of fibers transforming porous membrane into a dense membrane. In this study, various types of additives were tested to explore the appropriate solvent-rinsing process to obtain the maximum yield of CHF after drying and CM after carbonization. The effect of humidity on the drying process, as well as on the permeation properties of resulting carbon membranes were studied, and the process was optimized to obtain both high yield and permeation properties of carbon fibers.

The current research reports the development and optimization of a simple process to scale up the manufacturing of CHF precursor for CM. The information obtained from this research may be used to produce a limited number of CHF (laboratory scale) and several hundred fibers in one cycle (pilot-scale). The reported research discusses the design concept of the process to produce CHF, as well as the performance of resulting carbon hollow fiber membranes at pilot scale.

## 2. Materials and Methods

### 2.1. Materials

Acros Organics (Geel, Belgium) delivered the cellulose acetate (CA:MW 100,000, average acetyl content: 39.8%). *N*-methyl-2-pyrrolidone (NMP:purity > 99.5%) was purchased from Merck, Oslo, Norway. The additive polymer Polyvinylpyrrolidone (PVP:MW 10,000) was purchased from Sigma Aldrich, Oslo, Norway. Ionic exchanged water was used for coagulation and water wash process. Dimethyl sulfoxide (DMSO), Isopropanol, NaOH (>99%), and Glycerol (>98%) were purchased from VWR, Oslo, Norway.

### 2.2. Dope and Bore Solution Preparation and Filtration

**Figure 2:**
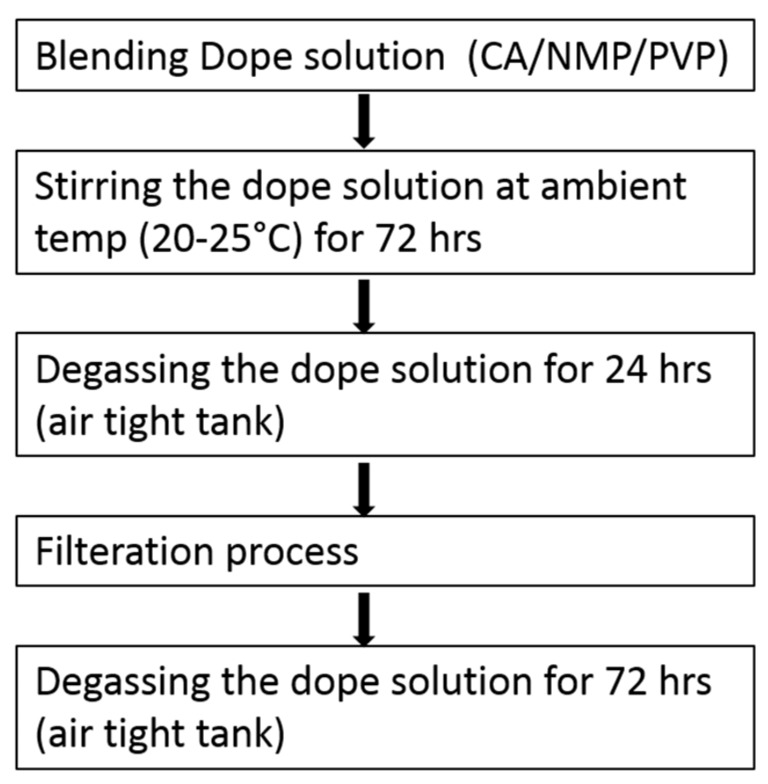
Steps involved in dope formation. CA, cellulose acetate; NMP, *N*-methyl-2-pyrrolidone; PVP, Polyvinylpyrrolidone.

The dope solution (20 kg in each batch) was prepared in a 50 L stainless steel mixing tank by adding 22.5 wt% of cellulose acetate (CA) and 5 wt% of PVP in dry state first and then 72.5 wt% of NMP solvent at room temperature (20–25 °C) was added before start stirring. Some spinning experiments were also performed by using DMSO solvent in the dope solution (replacing the NMP). After mechanical stirring for at least 72 h to ensure homogeneity of the dope solution, it was subsequently left to rest for 24 h to remove air bubbles before filtration process was started. By using 2 bara pressure of N_2_ and a gear pump, the dope solution was then forced through polypropylene melt blown filters into a storage tank. Then storage tank was held under a vacuum of −0.6 bara for 30 min to degas the dope solution before the vacuum was released. The dope solution was degassed at 1 atm by resting for at least three days after filtration prior usage. Figure 2 shows the steps followed during the dope formation process.

The bore solution was made in a bottle by adding a solvent (NMP or DMSO) and ionic exchanged water in the desired ratio, and then poured into the bore liquid storage vessel. The bore solution should preferably rest 24 h prior to use. A particle filter was installed in the bore line to avoid any contaminants in the bore solution.

### 2.3. Spinning of CA Hollow Fibers

**Figure 3:**
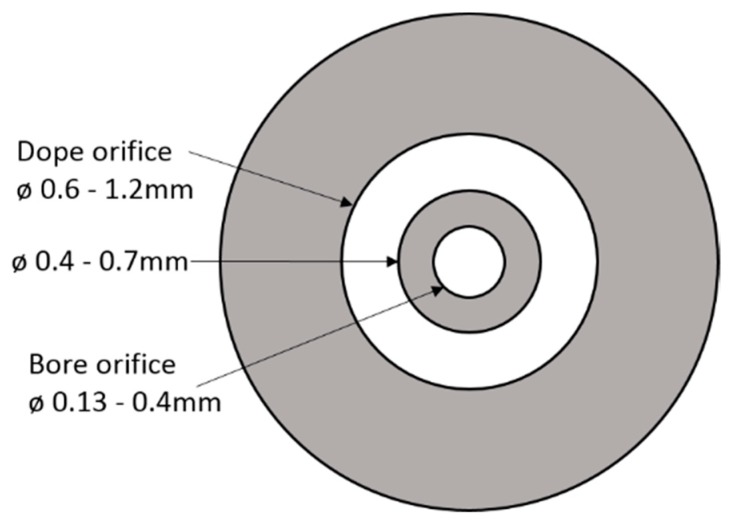
Spinneret type and range of different dimensions used for spinning of CAHF.

**Figure A1:**
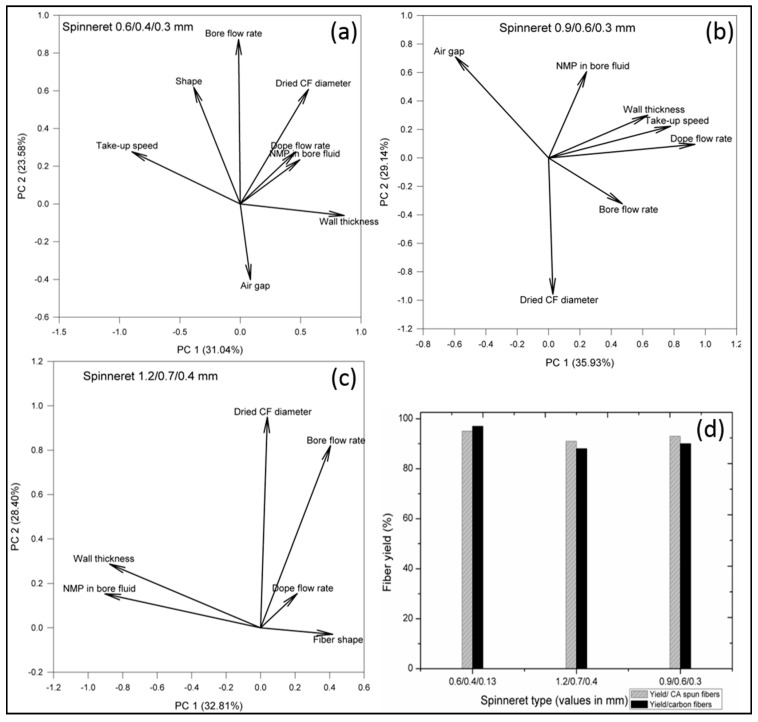
Principal component analysis (PCA) of three different types of spinnerets and resulting fiber yield.

A well-known dry/wet phase separation spinning process was used to spin the CA hollow fibers [19,24]. The dope solution and bore fluid were extruded through a tube-in-orifice jet type of spinneret to form a nascent hollow fiber. The extrusion rate of dope and bore fluid was controlled by two separate gear pumps, respectively. Spinnerets with three different sets of inner and outer dimensions (range is shown in Figure 3) were used to spin the hollow fibers. Principal component analysis (PCA) of three different types of spinnerets and resulting fiber yield (CHF and CM) are shown in Figure A1. Principal component analysis (PCA) of three different types of spinnerets and resulting fiber yield of Appendix A. The solvent would then evaporate into a dry environment for a short period (Air gap: 25 mm) before the fiber was immersed in a wet environment (water as external coagulant) in the coagulation bath. For each spinning-session, a continuous fiber length of (at least) 2.4 km was spun and rolled up in three layers on a collecting wheel. The rolled-up fiber was cut into 1200 fibers of 2 m length for each fiber. Altogether, more than 460 spinning-sessions of CA hollow fibers were spun with 50 batches of dope mixture, of which at least 100 sessions were spun by varying spinning conditions, e.g., spinneret dimension, air gap, extrusion rate, coagulation bath temperature, take up speed and different fiber collection methods to optimize the pilot scale production of CA hollow fibers. After optimizing the spinning process, the parameters were kept constant for the rest of the sessions, as shown in Table 1.

The partly coagulated fibers were guided by a wheel from the bottom of the coagulation bath to the godet-bath where some residence time was provided for solvent exchange before they were rolled up on a collection wheel.

#### 2.3.1. Fiber Collection Methods and Water Wash

Different types of collection methods were tried; however, only two methods that had a great effect on membrane morphology after deacetylation, drying and carbonization, are being reported here.

##### Squared Collection Wheel

**Figure 4:**
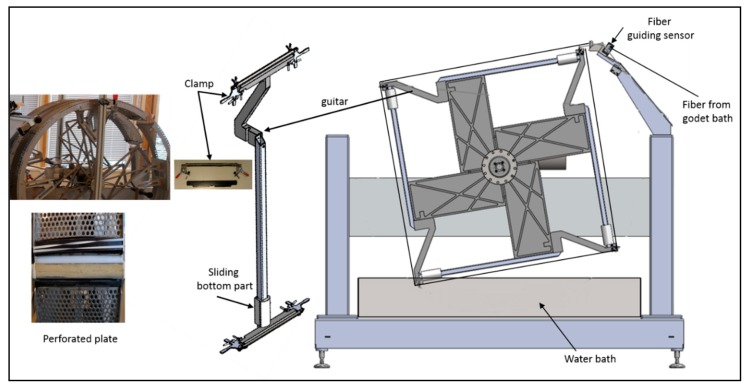
Collection wheel: Four fiber carriers attached to a rotating shaft (right side), perforated plate photos (left side).

Figure 4 presents the collection methods of CA hollow fibers. Four specially made stands “fiber carrier” were connected on a rotating shaft of the rig in such way that fibers were kept straight on each fiber carrier along the length and a sharp bending radius (avoiding the kink) was provided on the edges of the carrier. In order to promote the solvent exchange, a water bath with the continuous flow was present in such way that each fiber carrier was immersed in the bath while rotating on the shaft. After completion of the spinning process, clamps were applied on both sides/ends of the fiber carrier and fibers were cut (on the bends) with a sharp knife to separate the wheel into 4 individual carriers (nicknamed “Guitars”).

These fiber carriers applied some tension on the fibers, but they were designed to allow some contraction of the fibers to occur (sliding bottom part). The clamps on each end of the carrier were adjusted in such way that bore side of the fiber was open to exchange the solvent efficiently during water wash. Allowing some of the solvent exchange in a water bath, the wheel was left rotating for 2 h after clamping and cutting the fibers. The temperature of the water in the water bath was 10 °C. The fibers washed with 10 °C while on collection wheel for two hours and then in the water bath with continuously flowing water overnight gave the same properties as for 30 °C and 3 h wash. The water at 10 °C was easily available, therefore, most of the batches were washed overnight with cold water. Then fiber carriers were removed from the stand and placed in horizontal position in a water bath overnight so that all fibers were dipped in the bath, but not touching the bottom. A continuous flow (1.5 L/min) of fresh water was supplied to remove the solvent from the bath.

##### Circular Wheel with Perforated Collecting Plate

There is a trade-off between how long time the fiber stays on the wheel (aging by bore solution) and production efficiency, and this may determine the diameter of the collection wheel, the number of fiber layers or the take-up speed. An easy escape of water is favorable as it results in the suppression of macrovoids formation in the CA matrix. To enhance the efficiency of solvent exchange process during spinning process, two perforated circular plates (with the same width (25 cm) as the fiber carriers) were used to collect the fibers. Each plate was making half of the wheel’s circumference. A halfpipe made of acrylonitrile butadiene styrene (ABS) tubing (32 mm outer diameter (OD)) was attached to the gap between the perforated plates by 2 elastic bands (GUMA 120 × 8 mm). Foam was pre-fixed (glued in the halfpipe to conduct water). This halfpipe was placed at a level which prevents sharp bending of the collected fibers. A polycarbonate plate and an elastic band were used to make a loose clamp on the edges of each wheel half to ease the fiber endings and allow bore solution to exchange with water, as shown in Figure 4 (left hand side). The other half of the ABS pipe (made in a similar way) was placed on top of the fibers. Fibers were water-washed for 30 min in this way before clamping and moving them on the carriers. On completion of each spinning session, the fibers were transferred to two fiber carriers (2 m long each). Figure 4 shows the photo of such plates and fiber carriers. After clamping fibers on the carriers, they were placed in a horizontal bath for further water wash overnight to promote the removal of the bore solvent.

#### 2.3.2. Glycerol Wash

After water wash, the CA hollow fibers were soaked horizontally in glycerol solution overnight to remove (solvent exchange) the residual NMP in the fibers, and to prevent the largest pores and bore side from collapsing. Different concentrations of 5, 8, 10, and 15 vol% of glycerol in water were studied to see the effect on cellulose fibers, and ultimately carbon fibers, regarding shape and performance of the membrane. The glycerol solution was circulated by a pump in a cross-pattern (the inlet and outlet of the pump were immersed diagonally on opposite sides in the bath) to ensure good mixing and aid the solvent exchange process. The fiber carriers were immersed in the bath in such a way that all fibers were well below the liquid surface, but not touching the bottom of the bath.

#### 2.3.3. Deacetylation

The CAHF were then deacetylated with 90 vol% 0.075 M NaOH (Water) solution diluted with 10 vol% 2-propanol (isopropanol, IP) at ambient temperature for 2.5 h. Different concentrations of NaOH and dilutions of IP were tried, but it was observed that optimized degree of deacetylation of fibers was obtained by the mentioned composition. To improve mass transfer and homogeneous treatment of all fibers, liquid circulation via pumping and shaking of the bath and/or the fiber carriers were applied. It was experienced that the fibers that were not allowed to shrink during deacetylation could not give a CO_2_/N_2_ selectivity higher than 50 (CO_2_ permeability over 100 Barrer), however, fibers allowed to move along fiber carrier length and shrink (typically, 1–3%, during the deacetylation process) improved the permeation properties of the final carbon membrane. Hence the fibers were allowed to move without restriction during deacetylation. The reaction time, concentration of base and temperature were optimized. Two stirrers were used on both ends of the carriers and circulation pump to circulate the solution in a cross-pattern to avoid any stagnant regions for the solution in the bath.

During the deacetylation process, the contact between fibers and also the bottom of the bath should be minimized as it can inhibit the reaction and partial deacetylation may result. This can ultimately destroy the whole fiber quality by making that part brittle after drying. A shaking device was installed on the fiber carriers during deacetylation to avoid the contact between fibers and the bottom of the bath.

#### 2.3.4. Glucose Wash

Deacetylated fibers were immersed in an aqueous solution (T: 20 °C) of 7.5 wt% glucose for 30 min. The glucose solution was circulated using a pump in a cross-pattern to ensure good mixing. Direct drying after glucose wash resulted in sticky fibers. Therefore, fibers were immersed in fresh water (T: 10 °C) after glucose wash for 5 min to reduce the stickiness between the fibers.

#### 2.3.5. Drying

**Figure 5:**
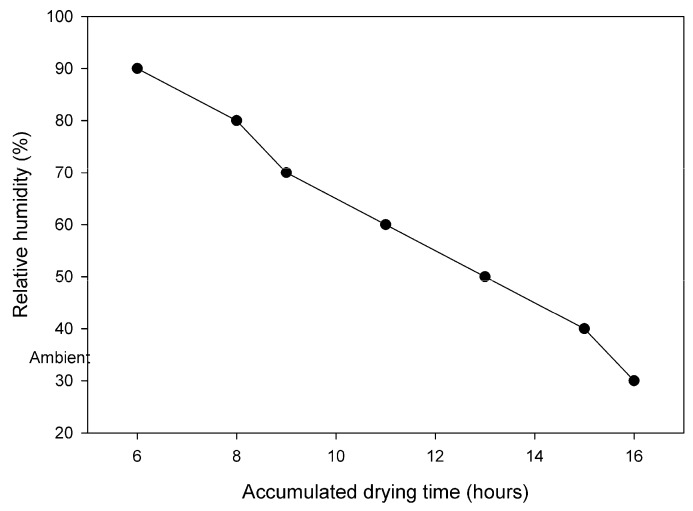
Accumulated time and humidity inside drying cabinet.

**Figure 6:**
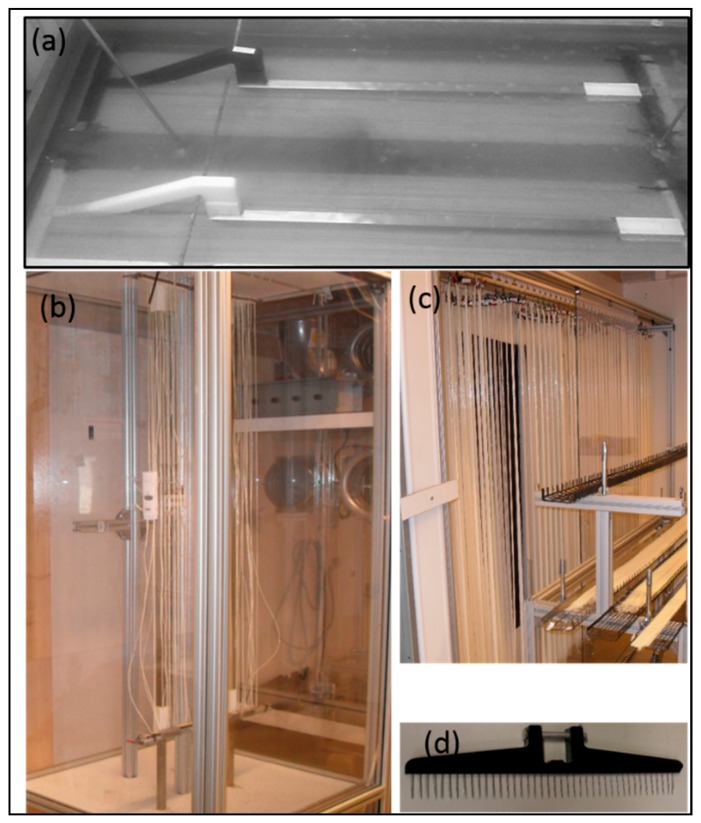
(**a**) Fibers lying in the deacetylation bath. (**b**) Fibers inside drying cabinet. (**c**) Hanging bundles of dry fibers and trolley with steel trays containing dry fibers. (**d**) Comb to split the fibers in equal bundles before drying.

Fibers were dried overnight at room temperature (20–25 °C) in a controlled humidity chamber, as shown in Figure 5. Figure 6 shows photos of fibers in various production states: (a) Fiber carriers during deacetylation, (b) fiber carriers inside drying cabinet, (c) hanging dry fibers and fibers ready for carbonization, and (d) comb to separate the fibers in equal bundles during drying. The fiber carriers were placed vertically (shown in Figure 6) inside the drying cabinet and the lock on the bottom end of the carrier was removed, allowing for shrinking of the fibers. Each fiber carrier contained 1200 fibers of 2 m length in three layers (400 fibers in each layer) and width of the total occupied surface on the carrier was 25 cm. A comb with 10 mm spacing between each tooth was used to further separate the sticky fibers (glucose effect) into equal groups, as the number of fibers in each bundle would determine the drying speed of that bundle. The effect of strand size on shrinkage of dried fiber was also studied. An extra load (averaged to 2.5 g per fiber) was added to the bottom end of the carrier. This acts as a counter force to the shrinking of the fiber clusters and assists in obtaining straight fibers. This extra load had a significant effect on the permeation properties of carbonized fibers, hence, the load was optimized in ordered to get straight fibers with good mechanical and acceptable permeation properties after carbonization.

A systematic investigation of the influences of drying parameters, such as humidity, extra load on the fibers, drying temperature was performed to obtain the straight cellulose fibers with high yield and good mechanical properties. Fibers were quality controlled after drying process by separating the broken, partially deacetylated, too curly, too brittle, and collapsed fibers from the good fibers. The good fibers were hung in bundles at ambient conditions. Storage temperature was reasonably stable, but RH was unstable due to seasonal change and no control of the internal environment. Number of days before loading the furnace for the carbonization process varied from 1–10.

#### 2.3.6. Measurements of Mechanical Properties of Fibers

Tensile strength and elongation at break of the fibers were measured after different production steps (spinning, water wash, deacetylation, and drying). No sophisticated instruments were available for the measurement of elongation at break and tensile strength of the fibers, therefore, some rough methods were used to get the estimated values for the mechanical properties of the fibers after each production step. For measurement of “elongation at break”, a 30 cm long fiber was cut after each production step. The fiber was stretched (medium: Air at room temperature) with fingers above a ruler to see the length when it breaks. Tensile strength was measured by using a cylindrical dynamometer. A single loop of fiber was made around the hook (medium: Air at room temperature) immediately after the production step, and then pulled it with fingers (independent of length of the fiber). The reported values for each fiber were from an average of fourteen fiber samples of seven spinning sessions.

### 2.4. Carbonization

A tubular horizontal furnace (Carbolite^®^, three zones split furnace) was used to carbonize the deacetylated hollow fibers. These deacetylated CA hollow fibers were carbonized at 550–650 °C under N_2_ or CO_2_ flow (0.7–1.9 L/min) using heating rate of 1 °C/min with several dwells and the final temperature of 650 °C for 2 h. Procedure details can be found in the patents [25,26]. The detailed procedure of the pilot-scale module construction for carbon membranes is reported elsewhere [14].

### 2.5. Permeation Testing

For the permeation experiments discussed here, CM (0.002 m^2^) modules were tested in a pilot scale “temperature and pressure rise” permeation set-up with shell side feed configuration. The system was constructed to tolerate medium range pressure single gas tests (CO_2_, N_2_, O_2_). The mass transport properties of CHF were measured with the single pure gases CO_2_ and N_2_ at 5 bar feed pressure and vacuum (0.1 bar) on the permeate side. Due to fire hazard limitations, CH_4_ was not tested at the membrane production facility. However, single gas CH_4_ and mixed gas (40% CO_2_ in CH_4_) experiments were performed in a dedicated field (set-up is described in [27]). The values for CH_4_ gas were obtained as: Selectivity αCO_2_/CH_4_ = 3 × αCO_2_/N_2_.

He et al. [13] has also performed the mixed gas experiments on carbon membrane (prepared with alike protocol) and results showed that the membrane performance for CO_2_ separation is the same or even higher in some cases for mixed gas as compared to single gas separation.

The performance of the membrane was evaluated by measuring the CO_2_ permeance in (m^3^(STP)/(m^2^·h·bar)) and CO_2_/N_2_ selectivities (α) using Equations (1) and (2). The tests were run continues from several hours to several days, to ensure that the transient phase of diffusion was passed, and a steady state obtained (*dp*/*dt* tends to a constant). The gas permeance, *P* (m^3^(STP)/m^2^·h·bar) was evaluated using the Equation (1):

(1) $$P=\frac{9.824.V\cdot (dp/dt)}{\Delta P\cdot A\cdot T_{exp}}.$$

Here, *V* is the permeate side volume (cm^3^) that should be measured using a pre-calibrated permeation cell as reported elsewhere [28,29]. However, the permeate side volume for this study was estimated by the tube length and cylinder volume on the permeate side. *dp*/*dt* and *A* are the collection volume pressure increase rate (mbar/s) and total active area of membrane (cm^2^) respectively, Δ*P* (bar) the pressure head and *T_exp_* (K) is the temperature for the experiment. The ideal selectivity was defined as the ratio of the pure gas permeances, as shown in Equation (2):

(2) $$\propto_{A/B}=\frac{P_{A}}{P_{B}}.$$

## 3. Results and Discussion

### 3.1. Effect of Spinning Parameters

Some initial observations during the spinning process were recorded when NMP was replaced by DMSO in the same ratio of the dope solution. For example, extrusion with DMSO as a solvent and bore fluid H_2_O/DMSO 15/85 *v*/*v*% gave higher uptake rate, which indicated greater porosity or less wall thickness. DMSO in bore fluid was also washed out faster than NMP, resulting in a firm and stronger fiber. It was observed that fibers spun at 25–30 °C in coagulation bath gave curly fibers after drying, however, applying 50 °C in the coagulation bath resulted in straight fibers after drying (free-hanging fibers). High temperature improved the mass transfer in coagulation bath and the time before polymer solidification became shorter.

After carbonization of fibers (650 °C, N_2_ 0.7–1.9 L/min), which were spun on the fiber carriers, they appeared to have an uneven surface and being more brittle than the fibers spun with NMP solvent. Permeance and selectivity were much lower. However, the carbonization of the fibers spun on the circular wheel with perforated plates resulted in a smooth surface and fibers being more flexible (could be looped down to 10 mm diameter). The permeance of these fibers was however so high (macrovoids) that there was no selectivity. Fu et al. [30] spun hollow fibers using a dope solution of DMSO/cellulose acetate butyrate and reported that fibers, when spun at high temperature (50 °C), had higher roughness on the outer surface. However, their membranes spun at 25 °C had a smooth outer surface, due to slow mass transfer rate.

**Figure 7:**
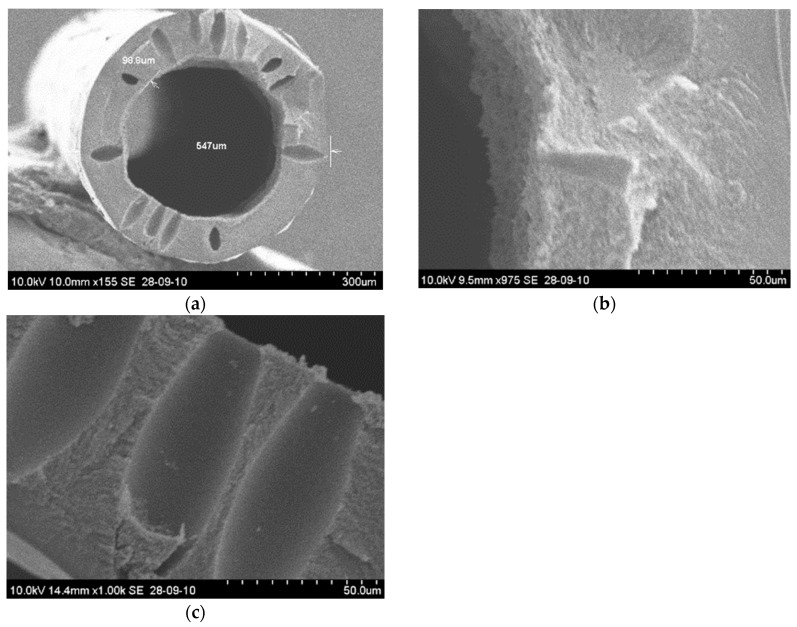
(**a**) CAHF cross section-72.5% dimethylsulfoxide (DMSO) in the dope solution; (**b**) Inner edge of the fiber; (**c**) Macro-voids on the wall magnified.

No further experiments were performed with DMSO in the dope solution. Figure 7 shows the scanning electron microscopic (SEM) images of the DMSO based CA hollow fibers. The macrovoids could easily be seen in the walls of the spun CAHF and these fibers did not have enough selectivity. It was observed that it is difficult to mend macrovoids during the carbonization process. Furthermore, chemical vapor deposition (CVD) was used to enhance the performance of prepared carbon membranes and it was concluded that fibers obtained using NMP had better properties compared to fibers prepared using DMSO.

**Figure 8:**
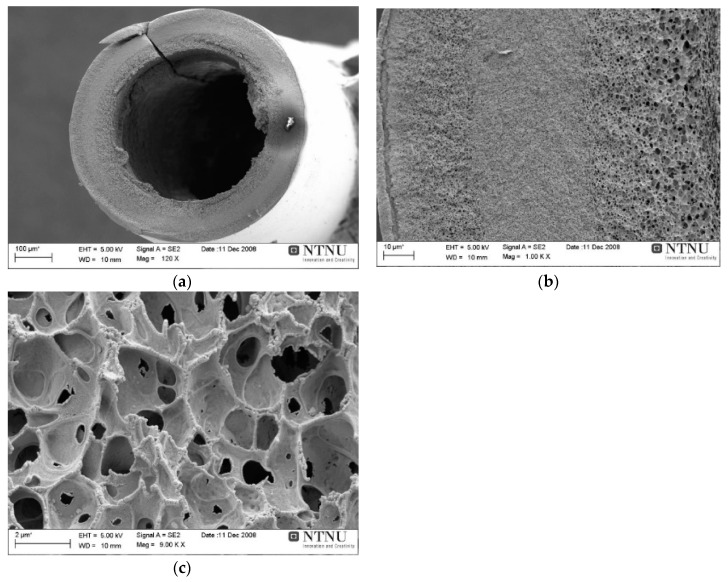
(**a**) CAHF cross-section-72.5% NMP in dope solution; (**b**) Wall magnified; (**c**) Wall inner edge magnified.

The rest of the batches were spun using NMP solvent in the dope solution. The CAHF spun with NMP gave high quality fibers with promising permeation properties. The SEM images of NMP based CAHF can be seen in Figure 8. The coagulation bath temperature of 25 °C resulted in the fiber wall more porous on the bore side. Pure water in the bore coagulant results in a thick and dense inner wall, which is not desired as the feed is introduced at the shell side of the fibers and the inner structure is only acting as a support. Bore solution containing up to 70% NMP have successfully been used, resulting in a porous lumen structure of the fiber, which is desired to minimize the gas transport resistance.

**Figure 9:**
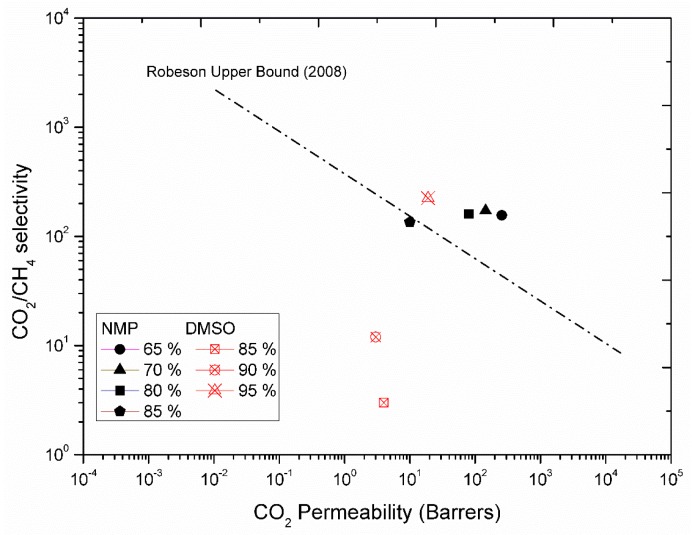
Effect of bore solvent and bore composition on gas permeation properties.

The effect of two different bore solvents and their compositions are presented in Figure 9. It can be seen that the membranes spun with 85% DMSO had lowest CO_2_ permeability with a CO_2_/CH_4_ selectivity of 4 as compared to 85% NMP, which had higher permeability, 10 Barrer, and selectivity of 135. Increase in concentration of DMSO improved the permeability of CO_2_, but the maximum value measured was 25 Barrer at 95% DMSO in bore fluid. Whereas membranes spun with bore solution containing 65 and 70% of NMP showed CO_2_ permeability of 256 Barrer and 144 Barrer with CO_2_/CH_4_ selectivity of 156 and 172 respectively, as shown in Figure 9.

**Figure A2:**
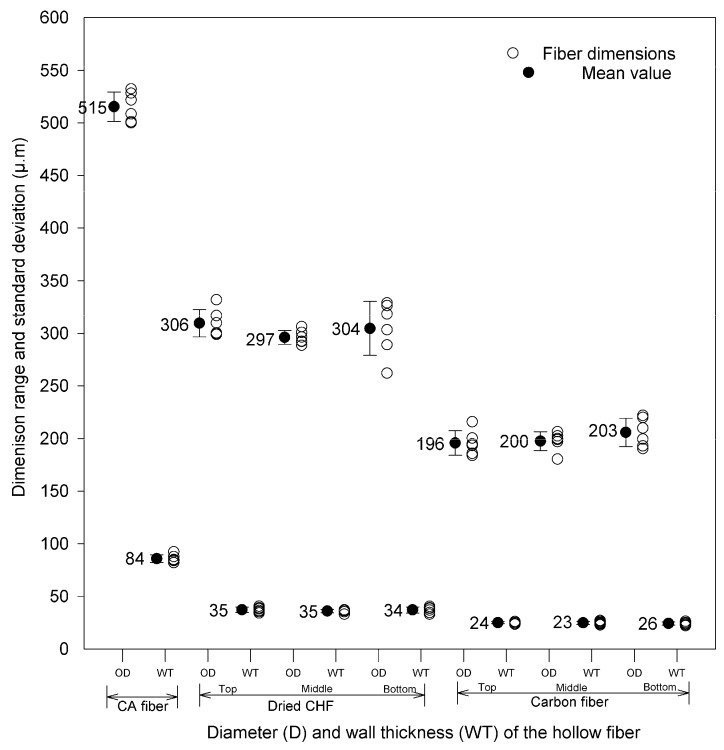
Outer diameter (OD) and wall thickness (WT) of CAHF, CHF, and CM at different positions of the fiber.

The outer diameter (OD) and wall thickness (WT) of some fiber samples from different spinning-sessions were measured. The length of the vertically hanging fibers were 2 m with the extra load on the bottom during drying, thus the variation in OD and WT of the fibers along fiber length was expected. The OD and WT of CHF and CM were measured at three different positions of the fiber: Top, middle, and bottom. Variation in the values and a mean value with standard deviation is shown in Figure A2 of Appendix A.

### 3.2. Effect of Water and Glycerol Treatment

To remove the solvent more efficiently with water, different methods were tried: Temperature, circulation with pump and stirring. It was observed that 3 h water wash at 30 °C with circulation gave good gas permeation properties of the membrane after carbonization.

Adsorbing the desired amount of glycerol into the pores of the fibers and increase the temperature (20 °C) helped to improve both the gas permeation properties, as well as mechanical properties of the carbon fibers. Fibers treated with 10 and 20% glycerol solution (circulating) gave carbon membrane with CO_2_ permeability below 20 Barrer and CO_2_/N_2_ selectivity of 40. Similar results were obtained when fibers were not treated with glycerol and in this case, most of the fibers were collapsed after the drying process. However, the CO_2_ permeability was above 200 Barrer with a selectivity of 50 for CO_2_/N_2_ when fibers were soaked in circulating 5 vol% glycerol aqueous solution (T: 20 °C) overnight.

### 3.3. Effect of Deacetylation

The rate of deacetylation depends on the diffusion velocity of OH^−^ ions inside the CAHF matrix and substitution reaction rate with an acetyl group. It is expected that the smaller the fiber, the faster the deacetylation. Fibers deacetylated for 2.5 h at ambient temperature gave good mechanical properties after drying and permeation properties above Robeson upper bound 2008 [31] after carbonization. Longer than 2.5 h deacetylation time resulted in a more morphological change in the pores of the fibers and decreased CO_2_ permeability after carbonization. The deacetylation parameters for similar kind of process at laboratory scale has been investigated by orthogonal experimental design in a recently published study [32]. Fibers deacetylated for less time than 2.5 h gave brittle and curly fibers after both deacetylation (yield after drying <50% of good fibers) and carbonization. It may be due to the partial deacetylation of CAHF. The fiber surface may be fully deacetylated, however, non/partially deacetylated inner part of the fiber may cause different drying rates on both surfaces which ultimately would result in curly or brittle fibers. The CO_2_ permeability of the subsequent carbon fibers was below 30 Barrer with a CO_2_/N_2_ selectivity of less than 10. Similar results regarding mechanical and permeation properties of fibers were obtained when deacetylation was conducted in aqueous solution or 5 vol% IP dilution of 0.075 M NaOH for 2.5 h. The surface of the fibers might be deacetylated, but the base molecules could not penetrate the fibers to hydrolyze the inner part. Hence, the resulting dried fibers were brittle and dense. In 90 vol% 0.075 M NaOH solution diluted with 10 vol% Isopropanol solution, the results indicated that the deacetylation was more homogenous and optimal for required permeation properties. This may be due to the swelling of CA with IP, providing a more open structure for the base to penetrate into the fiber. Liu et al. [20] have also observed that deacetylation in an aqueous solution of NaOH, appears to be complete on the fiber surface, making them hydroxyl-rich and leaving an acetyl-rich core. Whereas, in case of NaOH/EtOH solution, base molecules can penetrate the fiber to deacetylate the fiber more homogenously both on the surface and internally.

**Figure 10:**
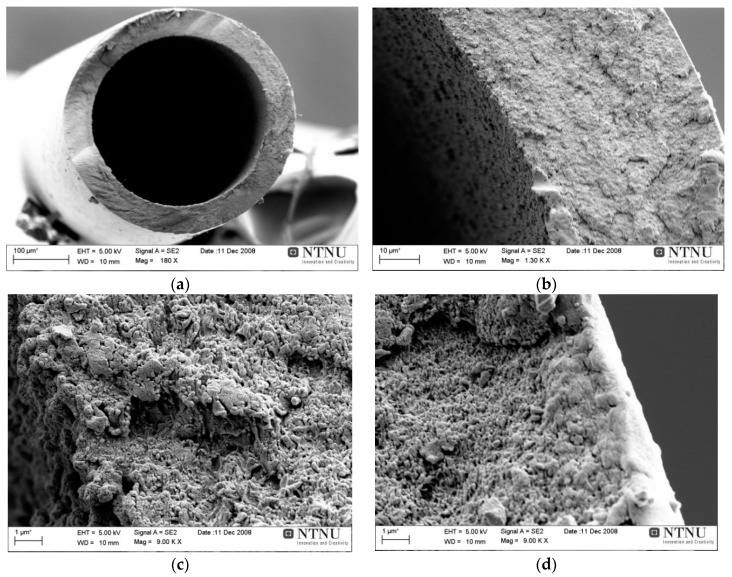
(**a**) Regenerated CHF after deacetylation of CAHF/cross section (**b**) wall magnified (**c**) wall inner edge magnified (**d**) wall outer edge magnified.

Due to the high sensitivity of the process, the attempts to lead an optimal deacetylation (instead of full) may deteriorate a uniformity and repeatability in cellulose fibers properties. There are too many parameters starting from non-uniformity of the initial cellulose acetate, external parameters as temp., mixing conditions etc., which may change from batch to batch or even within the same batch. This uncertainty leads to some uncontrolled final chemical composition. The deacetylation in 90 vol% 0.075 M NaOH solution diluted with 10 vol% Isopropanol solution provided dried cellulose fibers possessing good mechanical properties and yield was consistently over 80%. The SEM images of deacetylated hollow fiber are shown in Figure 10. These hollow fibers with optimized deacetylation gave stronger carbon fibers (loop with diameter: 8 mm) with permeation properties above Robeson upper bound 2008.

### 3.4. Effect of Glucose Wash

It was important to preserve and protect the natural micro porous structure of cellulose hollow fibers against irreversible collapse, which may occur during the drying process. Direct drying of deacetylated fibers caused the curliness and highest shrinkage (difference in length of fiber when treating with the additive solution and fiber length after drying) of hollow fiber membranes resulting in dense membranes with very low CO_2_ permeability. Jie et al. [24] also performed natural drying of cellulose fibers and they found that the membrane was too dense to give any gas permeation after drying.

**Figure A3:**
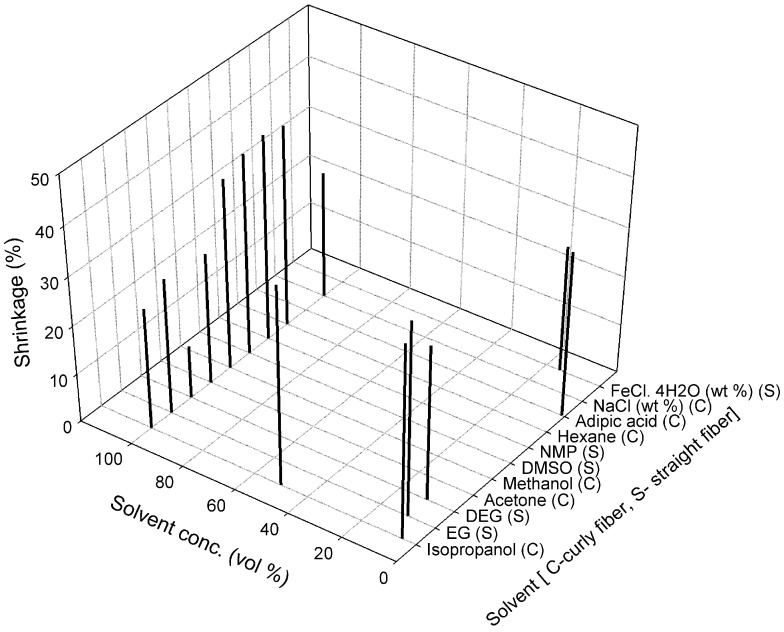
Effect of different solvent treatment (aqueous solutions) (after deacetylation) on shrinkage of dried cellulose fibers.

**Figure A6:**
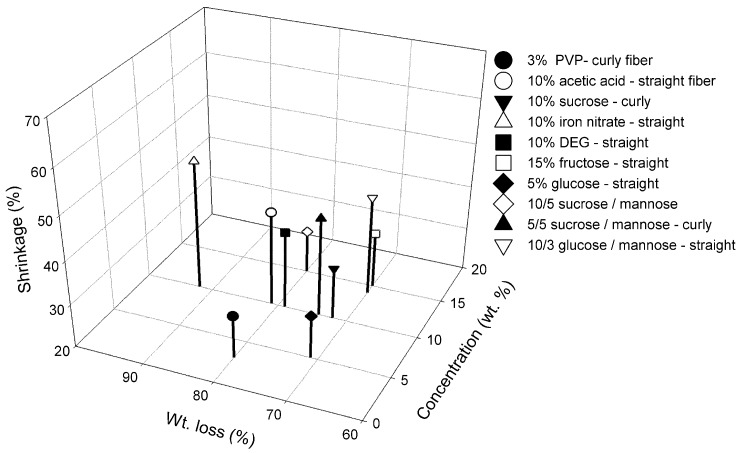
wt% loss and shrinkage after carbonization process when different additives were used after deacetylation.

It was found that cellulose fibers immersed in 7.5% glucose (aqueous) solution gives straight fibers with good mechanical properties after drying. Glucose molecules having suitable size entered most of the micro pores of the cellulose and was entrapped and engrafted easily. Glucose addition provided stiffness to the fibers and minimized the curliness, but had the disadvantage of making the fibers sticky when they were still wet. It might be possible to use some non-stick agents like those used in paper and food industry, but it will then be important that all traces of this product are then burnt off during carbonization and should not affect the gas permeation properties of the fibers. Nine different types of additives and eleven various types of solvents were used both individually and in combination with each other to study their effect on fiber shrinkage. Results are shown in Figure A3. Effect of different solvent treatment (aqueous solutions) (after deacetylation) on shrinkage of dried cellulose fibers—Figure A6 in Appendix A. Glycerol can also be used instead of glucose and it has similar effect on fibers after drying. However, it was observed that drying time increases when several fibers together on a carrier (instead of single fiber) were treated with glycerol. Glycerol with its high viscosity and boiling point accumulates on the bottom part of the fibers and it was hard to obtain homogenously dried fiber. The carbon fibers obtained after glucose treatment had good separation performance and mechanical properties. One theory may be that glucose (structure) behaves like cellulose during carbonization process, which results in carbon fibers with required properties.

### 3.5. Dry Fibers

The length of the fiber strands varied somewhat depending on their position on the collection wheel. Hence, when the fibers were put on the “fiber carrier” they may not all have the same level of initial tension applied to them.

**Figure 11:**
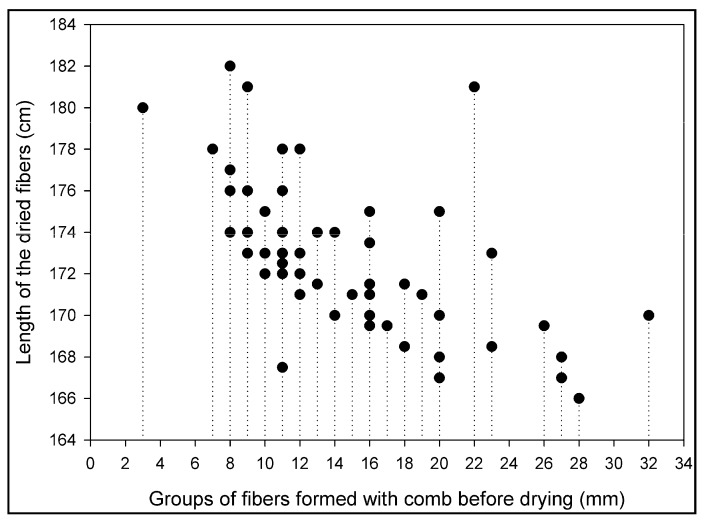
Drying effect on the bundle size of the cellulose fibers.

Dry cellulose fibers (fiber color, length, shape etc.) gave the first hint to how uniformly the fibers were processed. The effect of drying process (using the protocol shown in Figure 5) on different size of the bundles are shown in Figure 11. The results show that fibers with smallest bundle size gave the long and straight fiber after drying due to equal distribution of load at the bottom. The most likely explanation is that the smaller clusters of fibers dried quicker, hence more stretch. The fibers distributed in smaller than 10 mm bundle were longer, but collapsed in the bottom part of the fiber due to too much stress of the load. The bundle size was kept at almost 10 mm to enhance the yield of dried cellulose fibers.

**Figure 12:**
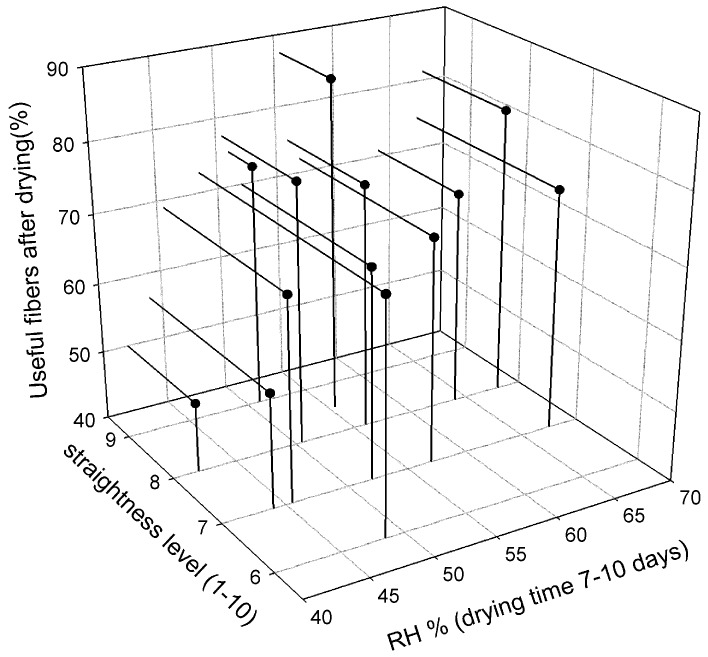
Effect of humidity on straightness and usefulness of the dried cellulose fibers. RH, relative humidity.

**Figure 13:**
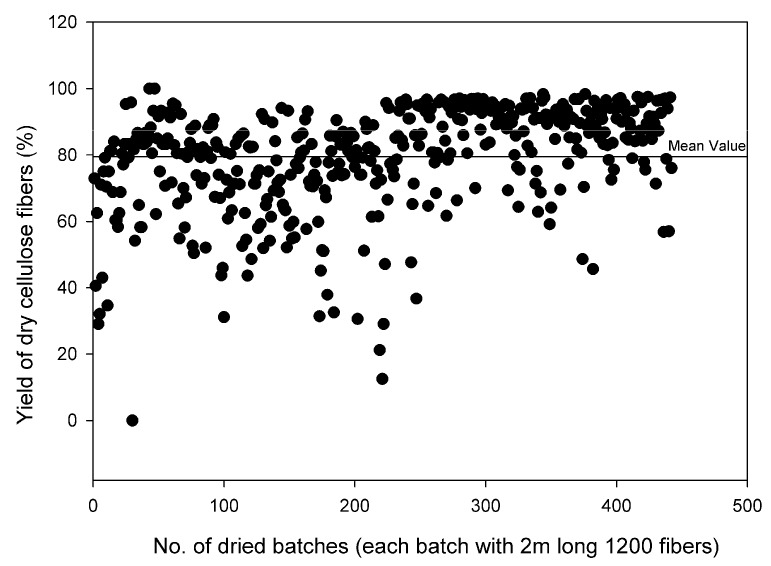
Yield (%) of 460 spinning-sessions of dried cellulose: RH (90–40%) and Temp (20–25 °C).

The effect of relative humidity (RH) is shown in Figure 12. The straightness was graded a visual level range from 1–10, where 10 was very straight fiber and 1 very curly fiber. Useful fibers were defined as those fibers, which could be used for further carbonization process. Figure 12 shows that RH during drying had a direct effect on the straightness and usefulness of the dried cellulose fibers. The fibers dried at RH over 50% at ambient temperature were straight and a high yield was obtained. It was observed that slow drying overnight with RH changing from 90 to ambient maximized the yield of the dried cellulose fibers. Figure 13 presents the yield of 460 spinning sessions of cellulose fibers dried using the drying protocol, as shown in Figure 5.

**Figure 14:**
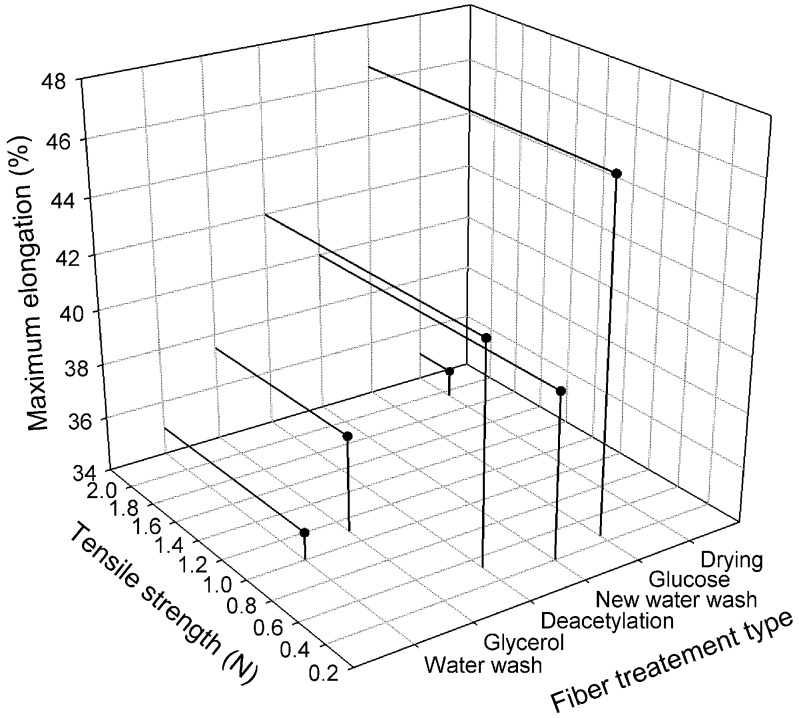
Mechanical properties of fiber after different production steps.

Mechanical properties of fibers after different production steps are shown in Figure 14. It shows that fibers after drying, possessed a maximum value of 1.9 N for tensile strength and minimum value 35% for elongation at break. Whereas the fibers after deacetylation, water wash, and glucose wash, showed minimum tensile strength, which was almost half of the value compared to the fibers just after spinning. The maximum value of elongation at break was measured after glucose treatment.

### 3.6. Carbonization and Gas Permeation Results

**Figure 15:**
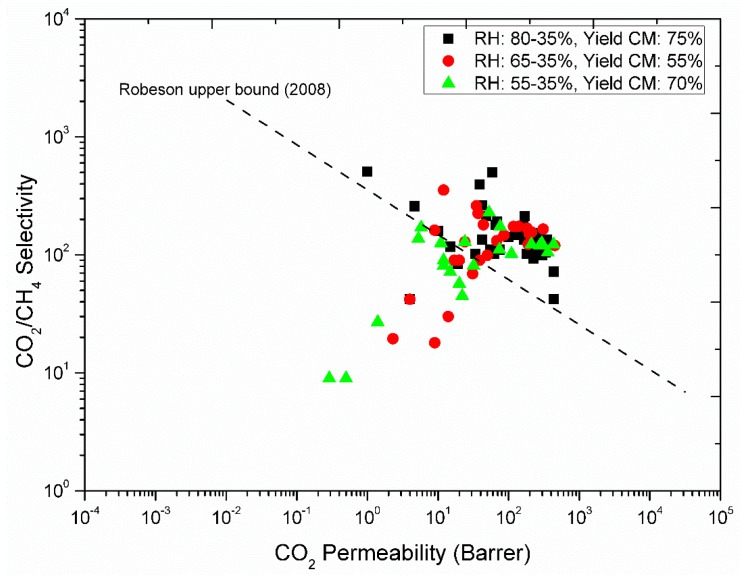
Gas permeation properties of carbon hollow fibers from regenerated cellulose precursor (RH: 80–35%; Black squares, RH: 65–35%; Red circles, RH: 55–35%; green triangles).

The final good carbon fibers should not be perfectly straight, but preferably having waves (i.e., wavelength > 10 cm) in order to (i) improve the gas flow pattern in the module, and to (ii) handle thermal expansion or shrinkage without breakage. However, curls (bends with a diameter less than 5 cm, i.e., ca 2× bundle diameter), kinks and loops must be eliminated. Gas permeation results of some batches dried at different RH are shown in Figure 15. These are small scale modules with an effective area of 0.002 m^2^ for each module. Details about pilot scale module construction, gas permeation performance and pore tailoring of the membranes after carbonization are reported elsewhere [14,33]. It was observed that fibers dried in RH: 65% → 35% when drying overnight at ambient temperature (23 °C) produced lowest number of carbon fibers. However, the gas permeation properties of these fibers were better compared to the fibers dried in RH: 55% → 35%. The maximum number of good fibers possessing both high gas permeation properties and mechanical properties were obtained at RH: 85% → 35%. These results indicate that slow drying of fibers at high RH humidity prevents the pore structure of dried cellulose fibers. It could be because along the cellulose fiber, water exists in two states: As bonded water (strong hydrogen bonds with cellulose molecules) and as free water (surrounded by the bonded water and no contact with the cellulose molecules). In the natural drying at lower RH: 55% → 35%, the water evaporated quite fast and the water-cellulose bonds were strong enough to pull the cellulose structure in a region with more dense/collapsed structure. However, in slow drying at higher RH: 85% → 35%, it could be that the hydrogen bonds of water-cellulose pulled the cellulose structure closer and closer until new hydrogen bonds between cellulose chains were formed, and keeping the pores structure stable as the free water evaporated gradually.

## 4. Conclusions

A pilot-scale system to produce regenerated cellulose hollow fiber membrane is reported in the current study. These regenerated CHF were used as precursors to produce high performance carbon membranes. Asymmetric CAHF with average outer diameter 515 µm and a wall thickness of 85 µm were spun in a dry/wet spinning process. The effect of DMSO and NMP as solvents in dope solution was studied. It was found that fibers spun with DMSO contain macrovoids on the wall. However, the fibers spun with NMP gave high quality fibers with promising (above Robeson upper bound 2008) gas permeation properties. The influence of DMSO and NMP as bore solvent on gas permeation properties was also reported. It was found that the increase in the concentration of DMSO improves the permeability of CO_2_, but the maximum value measured was 25 Barrer at 95% DMSO in bore fluid. Whereas, membranes spun with bore solution containing 65% and 70% of NMP showed CO_2_ permeability of 256 Barrer and 144 Barrer with CO_2_/CH_4_ selectivity of 156 and 172 respectively. The spun-CAHF underwent water wash and glycerol treatment to prevent the pores before they were transformed into regenerated cellulose through deacetylation process. It was found that fibers water washed overnight and then treated with 5% glycerol (aqueous) solution overnight give high performance carbon membranes with good mechanical properties. The deacetylation process was optimized by adjusting the different parameters (NaOH concentration, type of solvent, temperature, stirring, reaction duration, etc.) to achieve cellulose fibers with high yield on a pilot scale system. Optimized deacetylation of spun-CA hollow fibers (CAHF) was achieved by using 90 vol% 0.075 M NaOH aqueous solution diluted with 10 vol% Isopropanol for 2.5 h at ambient temperature. The number of collapsed and curly fibers were reduced with minimum shrinkage after drying process by the treatment of 7.5% glucose after deacetylation process. Drying conditions (temperature, relative humidity, rate of drying, stretch in fiber during drying) were optimized to achieve maximum (>95%) number of successful cellulose fibers. Relative humidity (RH) protocol for drying was improved after an investigation of different RH experiments. Separation performance results showed that RH changing from 80% to 35% at room temperature overnight gave maximum separation (above Robeson upper bound 2008) performance for the subsequent carbon hollow fibers. Tensile strength and maximum elongation of fibers were measured after each treatment. The pilot scale results showed that high performance carbon membranes can be made from regenerated cellulose (a relatively inexpensive precursor). This work identifies bottlenecks required for different unit operations in the preparation of a precursor for a carbon membrane with acceptable properties.

## Figures and Tables

**Table 1:** Optimized parameters for spinning, pre-treatment and carbonization processes.

Parameter	Values	Units
Dope Solution		
Composition	22.5 CA/5 PVP/72.5 NMP	wt%
flow rate	0.4	L/h
Temperature	RT (20–23)	°C
Bore Fluid		
Composition	30/35 H_2_O–65/70 NMP	vol%
flow rate	0.2	L/h
Temperature	RT (20–23)	°C
Other Parameters		
Air gap	25	mm
Coagulation medium/T	H_2_O/RT (20–23)	°C
Godet bath temperature	25–40	°C
Collection wheel	10	°C
Take up speed	14	m/min
Post-Treatment		
Water wash	10 °C	24 h
Glycerol wash	7.5 vol% in water (20 °C)	24 h
Deacetylation	0.075 M NaOH (aqueous sol.) diluted with 10 vol% IP	2.5 h
After wash	7.5 wt% glucose (20 °C)	30 min
Drying	T: 40–45 °C, RH: 90% → ambient	16 h
Carbonization		
Temperature	550–650 °C, 2 h soak	
Medium	N_2_ or CO_2_	0.7–1.9 L/min

**Figure A4:**
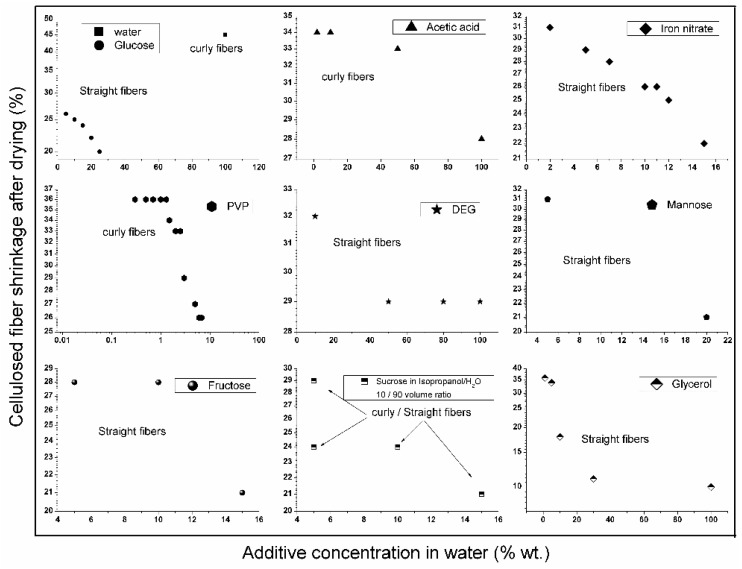
Effect of different additives (after deacetylation) on shrinkage of dried cellulose fibers.

**Figure A5:**
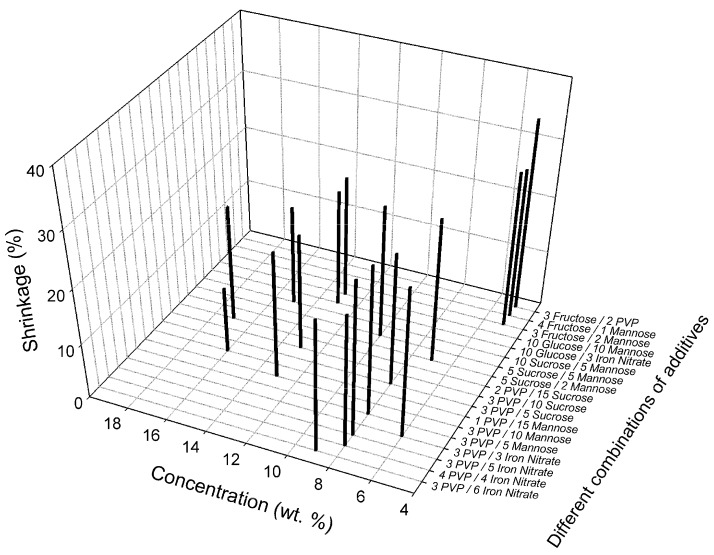
Combination of different additives (after deacetylation) effecting the shrinkage of dried cellulose fibers.
